# Safety, Effectiveness and Acceptability of the PrePex Device for Adult Male Circumcision in Kenya

**DOI:** 10.1371/journal.pone.0095357

**Published:** 2014-05-01

**Authors:** Paul J. Feldblum, Elijah Odoyo-June, Walter Obiero, Robert C. Bailey, Stephanie Combes, Catherine Hart, Jaim Jou Lai, Shelly Fischer, Peter Cherutich

**Affiliations:** 1 Clinical Sciences Unit, FHI 360, Durham, North Carolina, United States of America; 2 Nyanza Reproductive Health Society, Kisumu, Kenya; 3 Centers for Disease Control and Prevention/Kenya, Nairobi, Kenya; 4 University of Illinois at Chicago, Chicago, Illinois, United States of America; 5 Quantitative Sciences Department, FHI 360, Durham, North Carolina, United States of America; 6 National AIDS & STI Control Programme, Nairobi, Kenya; Johns Hopkins University Bloomberg School of Public Health, United States of America

## Abstract

**Objective:**

To assess the safety, effectiveness and acceptability of the PrePex device for adult medical male circumcision (MMC) in routine service delivery in Kenya.

**Methods:**

We enrolled 427 men ages 18–49 at one fixed and two outreach clinics. Procedures were performed by trained clinical officers and nurses. The first 50 enrollees were scheduled for six follow-up visits, and remaining men were followed at Days 7 and 42. We recorded adverse events (AEs) and time to complete healing, and interviewed men about acceptability and pain.

**Results:**

Placement and removal procedures each averaged between 3 and 4 minutes. Self-reported pain was minimal during placement but was fleetingly intense during removal. The rate of moderate/severe AEs was 5.9% overall (95% confidence interval [CI] 3.8%–8.5%), all of which resolved without sequelae. AEs included 5 device displacements, 2 spontaneous foreskin detachments, and 9 cases of insufficient foreskin removal. Surgical completion of MMC was required for 9 men (2.1%). Among the closely monitored first 50 participants, the probability of complete healing by Day 42 was 0.44 (95% CI 0.30–0.58), and 0.90 by Day 56. A large majority of men was favorable about their MMC procedure and would recommend PrePex to friends and family.

**Conclusions:**

The PrePex device was effective for MMC in Kenya, and well-accepted. The AE rate was higher than reported for surgical procedures there, or in previous PrePex studies. Healing time is longer than following surgical circumcision. Provider experience and clearer counseling on post-placement and post-removal care should lead to lower AE rates.

**Trial Registration:**

ClinicalTrials.gov NCT01711411

## Introduction

The World Health Organization (WHO), the Joint United Nations Programme on HIV/AIDS (UNAIDS), and other reproductive health organizations have recognized the protective effect of male circumcision in HIV acquisition [1]. Medical male circumcision (MMC) has been shown to reduce the incidence of HIV infection in men by about 60% [2]–[4]. Subsequent studies have confirmed the sustained protection of MMC against HIV acquisition by men [5]–[7].

Most MMC procedures are surgical; procedure times are approximately 15–30 minutes excluding anesthesia, involve suturing and control of bleeding, and can be associated with a variety of complications. Scale-up of adult MMC services could be accelerated by the availability of simplified non-surgical methods that could be done by non-physicians [1]. Although many devices are available and widely used for infant circumcision, there are fewer devices for adult circumcision and limited data on their effectiveness and safety. One such device is the PrePex™ Male Circumcision System, hereafter referred to simply as PrePex. Potential advantages of the PrePex device are that local injection anesthesia is not needed, no suturing is required, and placement and removal of the device are both quick.

Three clinical studies of PrePex have been conducted in Rwanda, with promising safety, effectiveness and acceptability results [8]–[10]. Based on these and other unpublished data, the WHO Technical Advisory Group on Innovations in Male Circumcision endorsed PrePex for use in Rwanda in 2012 [11], and subsequently added the PrePex device to its list of pre-qualified MMC devices for wider use [12]. The WHO also called for pilot implementation studies in countries seeking to scale-up MMC services to inform decision-making on the place of PrePex in national programs [13].

We conducted a study of the PrePex device in routine service delivery in western Kenya as part of the minimum package of HIV prevention services recommended by the Kenyan Ministry of Health (MOH) and the WHO, which includes HIV testing and counseling, exclusion of men with symptomatic sexually transmitted infection (STI) and provision of syndromic treatment as indicated, provision and promotion of condoms, and counseling on risk-reduction and safer sex.

## Methods

### Study design

Our prospective observational study of adult male circumcision with PrePex was done at sites in Nyanza Province, Kenya, where men seek voluntary medical male circumcision (VMMC) services. One site was at a fixed clinic, the UNIM Research and Training Center in the city of Kisumu. The other sites were dispensaries in Atela and Adiedo, more rural parts of Rachuonyo South and North districts respectively. Those facilities are visited by VMMC teams on a regular basis in what is termed outreach VMMC services.

The first 50 men were circumcised in the fixed Kisumu clinic and underwent intensive follow-up with six study visits (at Days 7, 9, 14, 28, 35, and 42 after device placement) to provide a detailed picture of safety and healing. An interim safety review of the first 50 participants was done by two outside evaluators with extensive MC experience, who recommended that the study continue as planned. The remaining 375 men were scheduled for two follow-up visits at 7 and 42 days after PrePex placement. Men were encouraged to return to the clinic at any time if they had problems or concerns. Men who were not completely healed by Day 42 were asked to return to the clinic weekly until healing was certified. We interviewed the men at each follow-up visit, and (with consent) took digital penile photographs of all four quadrants of the circumcision line to document adverse events, the course of healing and cosmetic appearance.

### Study eligibility

Mobilizers and peer educators informed community members living in close proximity to the three study sites about the availability of PrePex circumcision as a neutral alternative to the usual surgical procedure in the context of a research study. We then invited men who presented at the clinics to enroll in the study. Inclusion criteria were: ages 18 to 49 years; HIV-uninfected; in good general health and clinically free of STI; and provides contact information and written informed consent. A man was excluded from participation in the study if: his penis did not fit any of the five PrePex sizes; or he had a medical contraindication to MMC or study participation.

### Study objectives

The primary objective of this study was to assess the safety of PrePex MMC procedures during routine service delivery in Nyanza Province, Kenya. Per WHO recommendations [13], we measured the rate of moderate or severe adverse events (AEs), considering mild AEs to be within the normal spectrum of sequelae for any MC procedure. We categorized all circumcision-related AEs using the consensus PSI/WHO Adverse Event Action Guide [14] as modified in other studies of MC devices [15]. One modification defined moderate wound dehiscence to be a mucocutaneous gap greater than 1 cm along the shaft of the penis, between the edges of the wound; severe wound dehiscence was one that required surgical intervention. A second modification was our inclusion of self-reported moderate or severe pain in the AE classification. Participants were assessed for AEs at every follow-up visit for type, severity, seriousness and treatment (if any) of the event.

Secondary objectives of the study were to: determine the time to complete healing after PrePex placement, defined as a dry wound without any scab; evaluate the acceptability of PrePex procedures among clients and providers; estimate pain levels during PrePex procedures and wear; and compare outcomes in fixed versus outreach sites. We asked men about perceived pain at the end of PrePex placement; at 30 minutes post-placement; and at the removal visit immediately before, during and immediately after removal. We used a visual analog scale, with zero being no pain and ten the worst possible pain [16].

### PrePex device

The PrePex device is manufactured by CircMedTech Limited, is certified CE - Class IIa in the European Union, and has been approved by the U.S. Food and Drug Administration. PrePex is a sterile device consisting of an inner ring, elastic outer ring, placement ring, and verification thread; there are five sizes and proper fit is facilitated by a sizing accessory.

After sizing, the penis was disinfected and a circumcision line was marked on the outer surface of the prepuce approximately one centimeter proximal to the coronal sulcus. Lidocaine-containing anesthetic cream (10% concentration for the first 50 men and 2.5% with 2.5% prilocaine for the remainder) was applied to the glans penis and inner foreskin for lubrication and delayed analgesia. The elastic ring was loaded on to the placement ring, which was then placed at the base of the penis. The foreskin was held and stretched on each side with gauze pads, and the inner ring was inserted between the foreskin and glans penis and pushed down to the coronal groove. The placement ring was brought up to the circumcision line, and the elastic ring was deployed at the line and over the inner ring, securely compressing the foreskin between the two. Correct placement was verified, and the verification thread removed.

The man was requested to return 7 days after device placement for the removal procedure. Prior to removal, the necrotic foreskin was trimmed close to the inner ring using a special scissor supplied by the manufacturer, leaving a small rim of necrotized foreskin. The elastic ring was cut off using a surgical blade. The inner ring was then removed by hand or by using a spatula provided by the manufacturer. A non-adhesive dressing was applied after rinsing the penis with antiseptic solution.

Prior to the study, the experienced Kenyan MMC providers were trained by master trainers in Rwanda and certified proficient in: determining the suitability of clients for PrePex; correct PrePex sizing; the placement procedure; the removal procedure; post-removal follow-up; and AE management.

### Statistical considerations

By enrolling 425 men, we expected complete follow-up data on at least 400 men, per WHO guidelines for research on MMC devices [13]. The study size provided for 95% confidence intervals for observed AE rates of 4% or less.

We tabulated the frequency and percentage of men with moderate and severe AEs – overall, by type of service delivery site, and by cadre of MC provider. We tabulated the proportion of screening failures and reasons for exclusion; time needed for placement and removal procedures; and procedural and post-procedural pain. For the 50 men with enhanced follow-up, we evaluated time to complete healing using life table methods. For the full cohort, most of whom had scheduled follow-up visits only at Day 7 and Day 42, we simply calculated the proportion of men healed by Day 42, and the proportion healed later than Day 42. We set Day 0 to be the day of device placement. We summarized acceptability features, pain scores, time to return to normal activity, and satisfaction with the post-circumcision cosmetic results were summarized, along with the providers' opinions of the PrePex device.

### Ethics statement

The study was reviewed and approved by the IRBs of FHI 360 and the Kenya Medical Research Institute (KEMRI), as well as by the Kenyan Pharmacy and Poisons Board (PPB), which approved importation of the devices.

## Results

### Study population

A total of 464 men attending the clinics for VMMC were invited to join the study during HIV voluntary counseling and testing. 446 (96%) were enrolled, interviewed and examined. Of these enrollees, 432 (97%) had a PrePex device placed. The 14 men screened out (3.1% of enrollees) included eight (1.8%) with phimosis, hypospadias, narrow foreskin or other anatomic condition; three (0.7%) with symptomatic STI; one (0.2%) who withdrew consent; and two men for unspecified reasons. There were no men whose penis did not fit one of the five PrePex ring sizes. Four men under age 18 and one man without adequate consent documentation had PrePex devices placed; we excluded them from analysis, although they were followed for healing and safety in the same fashion as other participants. Thus, 427 men were in the analysis.

About one quarter of the procedures were done at the fixed UNIM clinic in Kisumu (N = 111), with the remainder at the two outreach health centers in Atela and Adiedo (N = 316). The overall median age of the participants was 20; the fixed-site cohort was older (median 26) than the outreach cohorts (median 19). Virtually all men were of the Luo ethnic group. About one quarter of participants reported no sexual intercourse in the past year. Of the sexually active men, approximately one quarter reported no condom use, and 44% reported always using condoms.

### Placement and removal procedures

Approximately two-thirds of all placement procedures were performed by nurses, and one-third by clinical officers, though this varied by type of service: 80% of fixed-site procedures were done by a clinical officer whereas 80% of outreach-site procedures were done by nurses. Nurses assisted in most procedures at both fixed and outreach sites. The mean and median durations of the placement procedures were 3.1 and 3 minutes respectively (from application of cream to cutting the verification thread), with little difference between the fixed and outreach sites. No medication was required during the procedures other than the routine anesthetic cream.

Removals were scheduled at seven days post-placement. Several early removals were done due to adverse events during device wear (see below). Removals also occurred in men with no AE on Days 5, 6, 8 or 9, all without sequelae. Mean and median removal times were 3.7 and 3 minutes respectively, nearly the same at the fixed and outreach sites. Half of the removals (inter-quartile range [IQR]) took 3 or 4 minutes.

### Pain assessments

Placement procedure pain was minimal, with a median score of 0 (IQR 0 to 2) on the 0–10 scale. Pain during the immediate post-placement period was even less, probably due to the delayed effect of the anesthetic cream inside the foreskin. Men reported more pain during removal, with mean and median pain scores of 5.3 and 5 and IQR of 4 to 6. That pain tended to be fleeting, usually at the moment of removal of the inner ring, with post-removal pain returning to a mean and median score of 1.6 and 2. Two-thirds of men reported return to usual activities on the day of the placement, and an additional 29% of men returned to usual activities the next day.

Self-reported mean and median pain scores at erection were 3.2 and 3 respectively. Scores were higher among men who had procedures done by clinical officers (mean 3.6) versus nurses (mean 2.9) (p<0.001).

### Adverse events

There were no AEs reported during the placement procedures. Remarkably, there were no infection-related AEs during the wearing of PrePex devices or after removal. A total of 29 moderate/severe AEs were reported among 25 men (5.9% of participants; 95% CI 3.8%–8.5%; Table 1). Among the first 50 participants who had enhanced follow-up and therefore greater opportunity to observe an event, there were eight AEs among five men (10% of participants); among the remaining 377 participants with two scheduled follow-up visits, there were 21 AEs among 20 men (5.3% of participants; two-sided p = 0.20). The final AE rate was higher in the fixed (9.0% of 111 men; 95% CI 4.4–15.9) than the outreach sites (4.7%; 95% CI 2.7–7.1; two-sided p = 0.11), and slightly higher among clinical officers (7.3%) than nurses (5.1%).

**Table 1:** Moderate and severe adverse events among all participants with device placement (N = 427), by type of site^1^.

	Fixed Site (N = 111)				Mobile Site (N = 316)				Total (N = 427)			
AE Onset Time/Classified AE Term^2^/Severity^3^	Num of Events	Num of Men	Percent of Men	Exact 95% CI	Num of Events	Num of Men	Percent of Men	Exact 95% CI	Num of Events	Num of Men	Percent of Men	Exact 95% CI
**MODERATE**												
… Device displacement	3	3	(2.7)	(0.6–7.7)	2	2	(0.6)	(0.1–2.3)	5	5	(1.2)	(0.4–2.7)
… Foreskin detachment	0	0	(0.0)	(0.0–3.7)	2	2	(0.6)	(0.1–2.3)	2	2	(0.5)	(0.1–1.7)
… Early device removal (before Day 7)	0	0	(0.0)	(0.0–3.7)	2	2	(0.6)	(0.1–2.3)	2	2	(0.5)	(0.1–1.7)
… Edema	2	2	(1.8)	(0.2–6.4)	0	0	(0.0)	(0.0–1.2)	2	2	(0.5)	(0.1–1.7)
… Excessive bleeding	1	1	(0.9)	(0.0–4.9)	1	1	(0.3)	(0.0–1.8)	2	2	(0.5)	(0.1–1.7)
… Insufficient skin removed	1	1	(0.9)	(0.0–4.9)	8	8	(2.5)	(1.1–4.9)	9	9	(2.1)	(1.0–4.0)
… Pain	3	3	(2.7)	(0.6–7.7)	0	0	(0.0)	(0.0–1.2)	3	3	(0.7)	(0.2–2.0)
… Wound dehiscence	1	1	(0.9)	(0.0–4.9)	0	0	(0.0)	(0.0–1.2)	1	1	(0.2)	(0.0–1.3)
… Poor appearance	1	1	(0.9)	(0.0–4.9)	0	0	(0.0)	(0.0–1.2)	1	1	(0.2)	(0.0–1.3)
**SEVERE**												
… Edema	2	2	(1.8)	(0.2–6.4)	0	0	(0.0)	(0.0–1.2)	2	2	(0.5)	(0.1–1.7)
**TOTAL**	14	10	(9.0)	(4.4–15.9)	15	15	(4.7)	(2.7–7.1)	29	25	(5.9)	(3.8–8.5)

**^1^Includes all events that are at least possibly related to the circumcision procedure.**

**^2^More than one AE could be recorded for a given participant.**

**^3^Maximum severity.**

Five device displacements occurred (1.2% of participants) and required surgical completion of the circumcision (Table 1). At least three and possibly all of the displacements were the result of attempted self-removals. Three men with device displacements had moderate/severe edema.

Spontaneous detachment of the foreskin while the device is in place is another AE particular to devices. Two such cases (0.5%) were observed in this study; both occurred late during the period of device wear and neither required any intervention beyond cleaning and dressing.

We observed two removals before Day 7 by clinic staff (0.5%) in men with early necrotic slough of foreskin tissue. In both cases, the provider performed surgical foreskin removal and healing was uneventful.

We recorded nine cases (2.1%) of insufficient skin removal (Table 1). Four of the nine men decided to have a surgical completion of the circumcision, while five men exited the study without surgical correction.

Other moderate/severe AEs included edema, pain, bleeding, and wound dehiscence.

### Time to complete healing

Time to healing is best evaluated among the first 50 men attending the fixed site, whose frequent follow-up allowed a more precise determination of the interval when healing took place. By Day 42, the probability of being completely healed was less than half (0.44, or 44 out of 100 men; 95% CI 0.30–0.58). This probability increased to 0.90 (95% CI 0.81–0.99) by Day 56 (Table 2). Estimated mean and median days to complete healing, taking censoring into account, were 48 and 49 respectively.

**Table 2:** Cumulative probability of complete wound healing for first 50 men, life table method^1^.

Fixed Site						
Interval during which certified wound healing occurred	Number with wound healing	n^2^	Cumu-lative proba-bility	S.e.	Lower 95% CI	Upper 95% CI
0–7 days	0	49.5	0	0	0	0
8–14 days	0	49.0	0	0	0	0
15–21 days	0	49.0	0	0	0	0
22–28 days	0	49.0	0	0	0	0
29–35 days	2	49.0	0.04	0.028	0.00	0.10
36–42 days	19	46.0	0.44	0.072	0.30	0.58
43–49 days	12	26.0	0.70	0.067	0.57	0.83
50–56 days	9	13.5	0.90	0.045	0.81	0.99
>56 days^3^	4	4.0	1	0	1	1

**S.e. = standard error; CI = confidence interval; mean days to complete healing = 48.0; median = 49 (range 35–82; interquartile range 42–51).**

**^1^From placement date to the date when complete healing is first recorded.**

**^2^Effective number in the interval.**

**^3^100% probability at >Day 56 interval reflects censoring at study exit as well as healing; assumes healing occurred at some point after the last contact.**

**Mean number of days to complete healing = 48.0; median = 49 (range 35–82; interquartile range 42–51) taking censoring into consideration.**

In the full cohort, half of the men were certified as completely healed by Day 42 (49.9%; 95% CI 45.0%–54.7%), with similar results at the fixed and outreach sites. This analysis makes the conservative assumption that men censored before Day 42 (4.2) were not healed by that time. An additional 26.7% of men were detected as healed later than Day 42, more at the fixed site (45.0%) than the outreach sites (20.3%). A large number of participants at the outreach sites (24.7%) were followed beyond Day 42 but exited the study without certification of complete healing. 4.2% of men were lost to follow-up at various times after device removal and before Day 42. Accounting for censoring, mean and median days to complete healing in the full cohort was estimated to be 48 and 44 (IQR 42 to 52), with estimates of mean and median of 47 and 44 days (IQR 42 to 50) at the fixed site, and 48 and 44 days (IQR 42 to 53) at the outreach sites.

There were 193 unscheduled follow-up visits made by 131 men, or 31% of participants. The majority of unscheduled visits (55%) occurred after Day 42 to monitor incomplete healing. Proportionately more unscheduled visits occurred at the fixed Kisumu clinic than the outreach clinics, possibly a function of easier access to that clinic.

### Post-MC abstinence and sexual activity

At every visit, men were asked about resumption of sexual activity, and the majority (88.1%) reported not having done so at any post-circumcision visit. Of the 49 men who had resumed sexual intercourse, 11 reported doing so before 42 days had elapsed. 65.3% of men who had resumed sex reported more pleasure during sex, while 6.1% reported less pleasure. 51% of men who had resumed sex had not used a condom. Just over 1% of men reported difficulties with erection at some point during the healing period.

### Acceptability parameters

In the Day 42 interview, each participant was asked open-ended questions regarding features of the PrePex device that he liked or disliked. The majority of men reported the procedure to be less painful than expected, and believed that it would improve penile hygiene (Table 3). 41% of the men stated that they were happy with their penile appearance. The most common dislikes, each mentioned by about one quarter of men, were odor while wearing the device, and pain during the removal procedure (Table 3). Almost a third of participants stated that there was nothing they disliked about the PrePex procedures. In general, men at the outreach sites were more likely to provide responses. Later in the interview, when men were asked specifically about penile appearance, virtually all stated that they were satisfied with the appearance of their penis, and would recommend PrePex MMC to male friends and family members (99% for both items).

**Table 3:** Selected features the participants liked and disliked about PrePex procedures.

	Delivery Service					
	Fixed Site (N = 111)		Outreach Sites (N = 316)		Total (N = 427)	
Feature	n^1^	(%)	n^1^	(%)	n^1^	(%)
**Liked about the circumcision**						
Less pain than expected	64	(58.7)	236	(75.6)	300	(71.3)
Improved personal hygiene	18	(16.5)	210	(67.3)	228	(54.2)
Happy with appearance of penis	11	(10.1)	160	(51.3)	171	(40.6)
Circumcision procedure was quick	33	(30.3)	117	(37.5)	150	(35.6)
Healthcare provider positive/friendly	37	(33.9)	94	(30.1)	131	(31.1)
No stitches	23	(21.1)	85	(27.2)	108	(25.7)
Nothing I liked	0	(0.0)	1	(0.3)	1	(0.2)
Total^2^	109	(100)	312	(100)	421	(100)
**Disliked about the circumcision**						
Bad odor while penis healing	16	(14.7)	98	(31.8)	114	(27.3)
Device removal too painful	15	(13.8)	87	(28.2)	102	(24.5)
More pain than expected	6	(5.5)	23	(7.5)	29	(7.0)
Pain/discomfort during erection	6	(5.5)	21	(6.8)	27	(6.5)
Nothing I disliked	42	(38.5)	89	(28.9)	131	(31.4)
Total^2^	109	(100)	308	(100)	417	(100)

**^1^N = number of men endorsing the feature in open-ended questions; more than one response possible.**

**^2^Denominators for percentages; the number of men who completed this section of the Interview Form.**

We also asked the PrePex MMC providers for their opinions and preferences regarding PrePex. All seven had performed at least 200 circumcisions by the forceps guided surgical method (FGM). Five of seven providers preferred PrePex over FGM and found it much simpler than surgical MC.

## Discussion

We observed moderate/severe adverse events in 5.9% of participants. Even excluding the 50 men with enhanced follow-up, 5.3% of men had a moderate/severe AE. This is higher than reported in the three published PrePex studies from Rwanda, in which the AE rates were 2% [8], 2.7% [9], and 0.8% of participants [10]. The rates in the Rwanda studies excluded expected MMC side effects, which if moderate could have been considered AEs in our study. A recent study in Uganda reported moderate/severe AEs in 1.6% of participants, including displacements and moderate bleeding, although the follow-up schedule and rate were not reported [17]. Our study nurtured a high index of suspicion for AEs in this initial use of PrePex in Kenya, incorporating: intensive follow-up of the first 50 participants; staff training to be vigilant about recording events; review of clinic notes during monitoring visits to determine if any AEs had been missed on the case report forms; strong encouragement that participants return to the clinic in case of any problems or concerns; and the addition of moderate/severe pain to the classification scheme. These features may help explain the higher AE rate in our study than earlier PrePex studies.

An active surveillance study in Nyanza Province following surgical MC found 7.5% of men reported treatment for post-MMC moderate/severe AEs. Men in that study were examined during home visits 28–42 days post-circumcision, so most problems would have already resolved and the AEs were largely self-reported [18]. Aside from that surveillance effort, AE rates following surgical MC have generally been lower than the rate we observed. The AE rate in our study is higher than those reported in high-volume service delivery settings at African sites using the forceps guided (Kisumu 1.8%; Orange Farm 1.8%) and dorsal slit and sleeve methods of surgical MC (Rakai 1.0%; higher with the latter) [19]–[21]. AE rates were intermediate in the landmark surgical MC trials for HIV prevention: 3.6% in Rakai, 1.5% in Kisumu and 3.8% in Orange Farm [18]. A recent study of another device for adult MMC, the ShangRing, reported a rate of moderate AEs of 1.0% with no severe AEs [22].

We found a higher AE rate in the initial 50 men than the remaining 378 men. There are three likely reasons for this. First, men lived close to that fixed clinic and had easier access than most participants at the outreach clinics. Second, the first 50 cases had more scheduled follow-up visits per participant, and greater opportunity to observe AEs. Third, there is an apparent training effect for optimal device placement and removal that prevailed in both the fixed site alone, and the combined outreach sites. At the outreach sites, the AE rate was 8.0% in the first 100 participants and 3.2% in the remaining 216 participants. This is a universal phenomenon that surgical MC programs have also experienced [19], [23]. The PrePex training effect may be minimized by applying lessons learned during our study.

The rate of PrePex device displacement (1.2%) was similar to one other study [17]. Displacement was not reported in the published Rwandan studies. The WHO TAG cited displacements in 0.2% of procedures [24]. Displacements can follow attempted self-removal, sexual activity/masturbation, erection, or accidental dislodgment, although we suspect that all the displacements in this study were self-induced. Depending on the timing during device wear, displacement can result in massive edema, blistering and pain, necessitating surgical completion of the circumcision. Clients should be counseled on the risks of tampering with the device or engaging in sexual activity while it is in situ. Programs must ensure that surgical back-up is readily available to handle such cases.

Surgical completion of PrePex MC was required in this study for two main reasons: device displacement or insufficient skin removal. Cases requiring surgical completion can be viewed as unsuccessful PrePex procedures. The total number of unsuccessful PrePex procedures in this study was nine (2.1%), following five displacements and four insufficient skin removals. (If we include the five men who had residual foreskin tissue but exited without surgery, the unsuccessful procedure rate would increase to 3.3%.) The percent of unsuccessful procedures in this study was higher than the 0.5% figure cited by WHO [25], and could be minimized by counseling patients to anticipate the necrosis process and manage pain using analgesics, rather than tampering with the device.

Our study did not compare MC techniques, but the time required for complete healing was meaningfully longer than that reported following surgical MC in Kenya, where 83.1% of men were completely healed by Day 35 and 94.1% of men were completely healed by Day 42 [26]. In our PrePex study, we observed a 0.90 probability of complete healing by Day 56 in the first 50 men, coinciding with the WHO statement that: “Healing following a device method was by secondary intention and took one to two weeks longer than with surgery” [24]. The Rwanda RCT reported that PrePex healing was 15 days longer than after surgical MC [9]. We were intentionally conservative in our certification of complete healing after PrePex MC: some of the men who required additional visits beyond Day 42 were quite close to being completely healed at Day 42. Longer healing time will be an added challenge for programs that integrate devices for MMC and counsel men to remain sexually abstinent until healing is complete [24].

Acceptability of PrePex MC was high, but several points deserve note and will inform future counseling in programs. Preventing odor during wear with a regimen of gentle penile irrigation, as is now recommended by the manufacturer, needs to be addressed explicitly with every patient. Pain management will be enhanced by specific messages about when pain is most likely to occur (anecdotally Days 2–3 and again around Day 5). Messages on wound management need to be precise; some men have mistaken the post-removal slough of residual foreskin tissue for infection and debrided the wound, resulting in pain, dehiscence, bleeding and delayed healing. The biggest challenge may surround the overall longer healing time by secondary intention needed for MMC with this device, and the difficulty of obtaining men's cooperation with longer abstinence and/or more consistent condom use.

This implementation study featured several notable strengths. Staff achieved a minimal loss to follow-up prior to Day 42, thanks to their experience and the presence of community mobilizers and tracers on staff. The study was large enough to yield reasonably precise estimates of moderate/severe AE rates. The 50-man safety evaluation with multiple follow-up visits offered a more precise estimate of the probability of healing by interval. We were able to compare endpoints between clinical officers and nurses, and between fixed and outreach services.

A weakness of the study related to loss of information: a large proportion of participants (24%) exited follow-up without certified healing. This occurred more frequently in the outreach sites which were in more rural areas where men resided further from the study clinics. Most were followed beyond Day 42, however, and so provided information on the proportion healed by that benchmark. That multiple clinics participated in the study was a strength, but one that allowed differential judgments of healing time by site. Interpretation of complete healing may be inherently subjective and can vary between MC providers and clinics [15]. Although unable to examine most participants at the outreach sites, the Site Investigator (EO-J) reviewed the penile photographs and case report forms and so mitigated differences in healing judgments.

In conclusion, the PrePex device was an effective method for adult male circumcision in fixed and outreach health facilities in Kenya. The method was well-accepted by the participants. We observed the reported advantages of the method, including: ease of task-shifting to lower cadres of providers; reduced procedure time; no need for injection anesthesia and suturing; and infrequency of infection.

In our view, the device's main drawbacks include: displacements requiring surgical completion and thus the continued need for back-up conventional surgery; insufficient skin removal requiring surgical correction; longer healing times; issues surrounding hygiene and odor while wearing the device; and wound care after removal. The adverse event rate was higher than for surgical MMC in Kenya, but all of the AEs resolved quickly without major consequences, and the AE rate diminished as providers gained more experience with the device. Appropriate training, along with clearer counseling messages on post-placement and post-removal care, should lead to lower AE rates.

## Supporting Information

File S1 **PrePex Classification of Adverse Events and Device Hazards Jan 2013**
(DOCX)

## References

[1] Samuelson J, Baggaley R, Hirnschall G (2013) Innovative device methods for adult medical male circumcision for HIV prevention: lessons from research. J Acquir Immune Defic Syndr 64: 127–129. 23892240 10.1097/QAI.0b013e3182a61dd3

[2] Auvert B, Taljaard D, Lagarde E, Sobngwi-Tambekou J, Sitta R, et al (2005) Randomized, controlled intervention trial of male circumcision for reduction of HIV infection risk: the ANRS 1265 Trial. PLoS Med 2 (11) e298. 16231970 10.1371/journal.pmed.0020298 PMC1262556

[3] Bailey RC, Moses S, Parker CB, Agot K, Maclean I, et al (2007) Male circumcision for HIV prevention in young men in Kisumu, Kenya: a randomised controlled trial. Lancet 369: 643–656. 17321310 10.1016/S0140-6736(07)60312-2

[4] Gray RH, Kigozi G, Serwadda D, Makumbi F, Watya S, et al (2007) Male circumcision for HIV prevention in men in Rakai, Uganda: a randomised trial. Lancet 369: 657–666. 17321311 10.1016/S0140-6736(07)60313-4

[5] Gray R, Kigozi G, Kong X, Ssempiija V, Makumbi F, et al (2012) The effectiveness of male circumcision for HIV prevention and effects on risk behaviors in a post-trial follow up study in Rakai, Uganda. AIDS 26: 609–615. 22210632 10.1097/QAD.0b013e3283504a3f PMC4296667

[6] Mehta SD, Moses S, Agot K, Odoyo-June E, Li H, et al (2013) The long-term efficacy of medical male circumcision against HIV acquisition. AIDS 27: 2899–2907. 23835501 10.1097/01.aids.0000432444.30308.2d

[7] Auvert B, Taljaard D, Rech D, Lissouba P, Singh B, et al (2013) Association of the ANRS-12126 Male Circumcision Project with HIV levels among men in a South African township: evaluation of effectiveness using cross-sectional surveys. PLoS Med 10 (9) e1001509. 24019763 10.1371/journal.pmed.1001509 PMC3760784

[8] Bitega JP, Ngeruka ML, Hategekimana T, Asiimwe A, Binagwaho A (2011) Safety and efficacy of the Prepex device for rapid scale-up of male circumcision for HIV prevention in resource-limited settings. J Acquir Immune Defic Syndr 58: 127–134. 21909032 10.1097/QAI.0b013e3182354e65

[9] Mutabazi V, Kaplan SA, Rwamasirabo E, Bitega JP, Ngeruka ML, et al (2012) Male circumcision comparison between a non-surgical device and a surgical technique in resource-limited settings: a prospective, randomized, non-masked trial. J Acquir Immune Defic Syndr DOI:10.1097/QAI.ob013e3182631d69 10.1097/QAI.0b013e3182631d69 22739133

[10] Mutabazi V, Kaplan SA, Rwamasirabo E, Bitega JP, Ngeruka ML, et al (2013) One-arm, open-label, prospective, cohort field study to assess the safety and efficacy of the PrePex device for scale-up of nonsurgical circumcision when performed by nurses in resource-limited settings for HIV prevention. J Acquir Immune Defic Syndr 63: 315–322. 23466648 10.1097/QAI.0b013e31828e6412

[11] World Health Organization (2012) Use of Devices for Adult Male Circumcision in Public Health HIV Prevention Programmes. WHO: Geneva.

[12] World Health Organization (2013) WHO Prequalification of Male Circumcision Devices Public Report. Product: PrePex; Number: PQMC 0001-001-00. WHO: Geneva.

[13] World Health Organization (2012) Framework for Clinical Evaluation of Devices for Adult Male Circumcision. WHO: Geneva.

[14] Rech D, Dickson K, Samkange C (2011) Adverse Event Action Guide for Male Circumcision. PSI and WHO: Washington, DC.

[15] Sokal DC, Li PS, Zulu R, Awori QD, Combes SL, et al (2014) Randomized controlled trial of the Shang Ring versus conventional surgical techniques for adult male circumcision: safety and acceptability. J Acquir Immune Defic Syndr 65: 447–455. 24583615 10.1097/QAI.0000000000000061

[16] Williamson A, Hoggart B (2005) Pain: a review of three commonly used pain rating scales. J Clin Nursing 14: 798–804. 10.1111/j.1365-2702.2005.01121.x 16000093

[17] Duffy K, Galukande M, Wooding N, Dea M, Coutinho A (2013) Reach and cost-effectiveness of the PrePex device for safe male circumcision in Uganda. PLoS One 8 (5) e63134. 23717402 10.1371/journal.pone.0063134 PMC3661578

[18] Herman-Roloff A, Bailey RC, Agot K (2012) Factors associated with the safety of voluntary medical male circumcision in Nyanza province, Kenya. Bull World Health Org 90: 773–781. 23109745 10.2471/BLT.12.106112 PMC3471059

[19] Krieger JN, Bailey RC, Opeya JC, Ayieko BO, Opiyo FA, et al (2007) Adult male circumcision outcomes: experience in a developing country setting. Urol Int 78: 235–240. 17406133 10.1159/000099344

[20] Lissouba P, Taljaard D, Rech D, Doyle S, Shabangu D, et al (2010) A model for the roll-out of comprehensive adult male circumcision services in African low-income settings of high HIV incidence: the ANRS 12126 Bophelo Pele Project. PLoS Med 7 (7) e1000309. 20652013 10.1371/journal.pmed.1000309 PMC2907271

[21] Buwembo DR, Musoke R, Kigozi G, Ssempiija V, Serwadda D, et al (2011) Evaluation of the safety and efficiency of the dorsal slit and sleeve methods of male circumcision provided by physicians and clinical officers in Rakai, Uganda. BJU Int 109: 104–108. 21627752 10.1111/j.1464-410X.2011.10259.x PMC4326085

[22] Kigozi G, Musoke R, Watya S, Kighoma N, Ssebbowa P, et al (2013) The acceptability and safety of the Shang Ring for adult male circumcision in Rakai, Uganda. J Acquir Immune Defic Syndr 63: 617–621. 23614991 10.1097/QAI.0b013e3182968dda PMC3805675

[23] Kiggundu V, Watya S, Kigozi G, Serwadda D, Nalugoda F, et al (2009) The number of procedures required to achieve optimal competency with male circumcision: findings from a randomized trial in Rakai, Uganda. Br J Urol Intl 104: 529–532. 10.1111/j.1464-410X.2009.08420.x PMC2748867 19389002

[24] World Health Organization (2013) Guideline on the Use of Devices for Adult Male Circumcision for HIV Prevention. WHO: Geneva. 24624479

[25] World Health Organization (2013) Meeting Report: WHO Technical Advisory Group on Innovations in Male Circumcision: Evaluation of Two Adult Devices. WHO: Geneva.

[26] Rogers JH, Odoyo-June E, Jaoko W, Bailey RC (2013) Time to complete wound healing in HIV-positive and HIV-negative men following medical male circumcision in Kisumu, Kenya: a prospective cohort study. PLoS One 8 (4) e61725. 23613918 10.1371/journal.pone.0061725 PMC3626701

