# Supplementary material for: Safety, Effectiveness and Acceptability of the PrePex Device for Adult Male Circumcision in Kenya
Source: PLoS One. 2014 May 1;9(5):e95357. doi: 10.1371/journal.pone.0095357 (PMC4006910; doi:10.1371/journal.pone.0095357)
Supplement: File S1 — PrePex Classification of Adverse Events and Device Hazards Jan 2013 (DOCX) [file pone.0095357.s001.docx]

Rev - 19^th^ June 2012

**PrePex MC**

**Classification of Adverse Events and Device Hazards**

| **Adverse**  **Event** | **Description** | **Severity** | **Code** |
| --- | --- | --- | --- |
| **A. During Placement** | | | |
| Pain | Pain score of 8 or more not requiring anesthesia  Requires anesthesia  Not controlled by additional anesthesia | Mild  Moderate  Severe | APP1  APP2  APP3 |
| Difficulty in applying the device | Could not apply device; determined as contra indicated; no harm to tissue or subject  Had to push unusually hard, but no harm to tissue or subject and no change to procedure  The device cut through the foreskin, with or without minor bleeding, No change in procedure  The device cut through the foreskin, with or without minor bleeding, and requiring a change to surgical method  The device cut through the foreskin, causing significant bleeding and requiring a change to surgical method | No AE  No AE  Mild  Moderate  Severe | --  --  ADD1  ADD2  ADD3 |

| **Adverse**  **Event** | **Description** | **Severity** | **Code** |
| --- | --- | --- | --- |
| **B. While Wearing the Device** | | | |
| Pain / Discomfort | Not requiring intervention beyond painkiller or anesthetic cream  Requiring early device removal by PrePex Operator or by client  Requiring early device removal and anesthesia  Not controlled by device removal or additional anesthesia | Not AE  Mild  Moderate  Severe | --  BWP1  BWP2  BWP3 |
| Device displacement / spontaneous detachment | Device displacement with no clinical consequences  Complete spontaneous detachment, or patient removed device himself with no adverse clinical consequences  Device displacement requiring re-placement  Device displacement requiring surgical intervention  Displacement or detachment and penile damage present | Not AE  Mild  Moderate  Severe | --  BDD1  BDD2  BDD3 |
| Early device removal (i.e. 4 days or less with the device) | Device removed due to pain, swelling or bleeding  Device removed due to pain, swelling or bleeding requiring surgical intervention  Device removed and penile damage present | Mild  Moderate  Severe | BER1  BER2  BER3 |
| Edema | More edema than usual but not causing any discomfort to the patient  Moderate edema causing the patient discomfort, though managed with conservative measures  Severe edema, causing the patient discomfort, uncontrolled with conservative measures | Mild  Moderate  Severe | BED1  BED2  BED3 |
| Hematoma | Mild contained hematoma, not requiring any treatment  Hematoma requiring surgical drainage/exploration but no evidence of active bleeding  Rapidly expanding hematoma suggesting active bleeding requiring surgical exploration or referral | Mild  Moderate  Severe | BHM1  BHM2  BHM3 |

| **Adverse**  **Event** | **Description** | **Severity** | **Code** |
| --- | --- | --- | --- |
| **C. During Device Removal** | | | |
| Pain | Pain score of 6 or less lasting for less than 2 minutes  Pain score of 8 or more lasting for over 2 minutes  Requires anesthesia  Not controlled by additional anesthesia | Not AE  Mild  Moderate  Severe | --  CPR1  CPR2  CPR3 |
| Excessive bleeding | More bleeding than usual, but easily controlled  Bleeding that requires suture to control  Blood transfusion or transfer to another facility for management required | Mild  Moderate  Severe | CBL1  CBL2  CBL3 |
| Edema | More edema than usual but not causing any discomfort to the patient  Moderate edema causing the patient discomfort, though managed with conservative measures  Severe edema, causing the patient discomfort, uncontrolled with conservative measures | Mild  Moderate  Severe | CED1  CED2  CED3 |
| Hematoma | Mild contained hematoma, not requiring any treatment  Hematoma requiring surgical drainage/exploration but no evidence of active bleeding  Rapidly expanding hematoma suggesting active bleeding requiring surgical exploration or referral | Mild  Moderate  Severe | CHM1  CHM2  CHM3 |
| Infection | Pain and erythema with no obvious swelling  Painful swelling with erythema or elevated temperature or purulent wound discharge  Cellulitis or wound necrosis | Mild  Moderate  Severe | CIN1  CIN2  CIN3 |
| Device removal difficulties | Difficult removal, with pain score of 8 or more lasting for over 2 minutes  Difficult removal, with abrasion of shaft or glans  Difficult removal, requiring injection of local anesthetic or requiring up to three sutures post-removal  Difficult removal, requiring more than three sutures  Difficult removal, with penile damage | Mild  Mild  Mild  Moderate  Severe | CDR1.1  CDR1.2  CDR1.3  CDR2  CDR3 |
| Damage to the penis | Mild bruising or abrasion, not requiring treatment  Bruise or abrasion to the glans or shaft of the penis requiring pressure dressing or surgery to control  Portion or all of the glans or shaft of the penis severed | Mild  Moderate  Severe | CDP1  CDP2  CDP3 |

| **Adverse**  **Event** | **Description** | **Severity** | **Code** |
| --- | --- | --- | --- |
| **D. Within 6 Weeks Post Removal** | | | |
| Pain | Symptoms of pain requiring bed rest for less than half the day  Pain requiring bed rest for more than half day  Excruciating pain requiring total bed rest | Mild  Moderate  Severe | DPA1  DPA2  DPA3 |
| Excessive bleeding | More bleeding than usual, but easily controlled  Bleeding that requires suture to control  Blood transfusion or transfer to another facility for management required | Mild  Moderate  Severe | DBL1  DBL2  DBL3 |
| Edema | More edema than usual but not causing any discomfort to the patient  Moderate edema causing the patient discomfort, though managed with conservative measures  Severe edema, causing the patient discomfort, uncontrolled with conservative measures | Mild  Moderate  Severe | DED1  DED2  DED3 |
| Hematoma | Mild contained hematoma, not requiring any treatment  Hematoma requiring surgical drainage/exploration but no evidence of active bleeding  Rapidly expanding hematoma suggesting active bleeding requiring surgical exploration or referral | Mild  Moderate  Severe | DHM1  DHM2  DHM3 |
| Infection | Pain and erythema with no obvious swelling  Painful swelling with erythema or elevated temperature or purulent wound discharge  Cellulitis or wound necrosis | Mild  Moderate  Severe | DIN1  DIN2  DIN3 |
| Damage to the penis | Mild bruising or abrasion, not requiring treatment  Bruise or abrasion to the glans or shaft of the penis requiring suture or surgery to control  Portion or all of the glans or shaft of the penis severed | Mild  Moderate  Severe | DDP1  DDP2  DDP3 |
| Delayed wound healing | Healing takes longer than usual, but no extra treatment necessary  Additional non-operative treatment required  Requires re-operation to correct | Mild  Moderate  Severe | DDW1  DDW2  DDW3 |
| Appearance | When healing is complete, subject concerned, but no discernable deformity  When healing is complete, minimal deformity does not require re-operation  Significant deformity requires re-operation to correct | Mild  Moderate  Severe | DAP1  DAP2  DAP3 |
| Problems with voiding | Transient complaint by subject that resolves without treatment  Requires a special return to the clinic, but no additional treatment  Requires referral to another facility for management | Mild  Moderate  Severe | DVO1  DVO2  DVO3 |

| **Adverse**  **Event** | **Description** | **Severity** | **Code** |
| --- | --- | --- | --- |
| **E. Six Weeks or More Post Removal** | | | |
| Infection | Pain and erythema with no obvious swelling  Painful swelling with erythema or elevated temperature or purulent wound discharge  Cellulitis or wound necrosis | Mild  Moderate  Severe | EIN1  EIN2  EIN3 |
| Delayed wound healing | Healing takes longer than usual, but no extra treatment necessary  Additional non-operative treatment required  Requires re-operation to correct | Mild  Moderate  Severe | EDW1  EDW2  EDW3 |
| Appearance | Subject concerned, but no discernable deformity  Minimal deformity does not require re-operation  Significant deformity requires re-operation to correct | Mild  Moderate  Severe | EAP1  EAP2  EAP3 |
| Excessive skin removed | Client concerned, but there is no deformity on erection  Causes slight discomfort on erection but surgical correction not necessary  Interferes with sexual life and surgical correction is necessary | Mild  Moderate  Severe | EES1  EES2  EES3 |
| Insufficient skin removed | Prepuce partially covers the glans only when extended  Prepuce still partially covers the glans and re-operation is required to correct  Not applicable | Mild  Moderate  Severe | EIS1  EIS2  EIS3 |
| Torsion of penis | Torsion is observable, but does not cause pain or discomfort  Causes mild pain or discomfort on erection, but additional operative work not necessary  Requires re-operation or transfer to another facility to correct the problem | Mild  Moderate  Severe | ETP1  ETP2  ETP3 |
| Erectile dysfunction | Client reports occasional inability to have an erection  Client reports frequent inability to have an erection  Client reports complete or near complete inability to have erections | Mild  Moderate  Severe | EED1  EED2  EED3 |
| Psycho-behavioural problems | Client reports mild sexual dissatisfaction attributed to male circumcision, but no significant psycho-behavioral consequences  Client reports significant sexual dissatisfaction attributed to male circumcision, but no significant psycho-behavioral consequences  Significant depression or other psychological problems attributed by the participant to the male circumcision | Mild  Moderate  Severe | EPB1  EPB2  EPB3 |
| Other AEs | Other AEs are described below* | -- |  |

**Other AEs**

- Liver or pancreatic abnormalities;
- Neurologic conditions of the central nervous system (e.g., meningitis, encephalitis, convulsions and headaches, visual and auditory disturbances, strokes), and peripheral neurologic conditions (e.g., peripheral neuropathies, motor weakness etc.);
- Myocarditis and pericarditis;
- Dermatologic condition affecting the genital, pubic or perianal areas or groin; and,
- Injuries and accidents.

Common illnesses unrelated to male circumcision will not be included in the “other AE” category to avoid unnecessary reporting of irrelevant events. Such AEs include the following:

- Malaria and other common parasitic infections;
- Gastrointestinal (GI) tract diseases, including diarrhea, gastroenteritis (bacterial, viral or parasitic), and other GI conditions, and oropharangeal infections;
- Respiratory illnesses (Upper Respiratory Tract Infections, acute lower respiratory infection, pleurisy), including tuberculosis, influenza or other respiratory infections;
- Angina, myocardial infarction, congestive cardiac failure;
- Dermatologic conditions (e.g., scabies, infected and uninfected rashes, pruritis, fungal infection), except those affecting the genital, pubic or perianal areas or groin
- Neoplasms not affecting the genitourinary tract; and,
- Surgical conditions not related to the intervention (e.g., hernias) and which do not require hospitalization.
